# Visible-Light-Enhanced Lanthanide Heterostructure with Synergistic Antibacterial and Anti-inflammatory Activity to Accelerate Wound Healing

**DOI:** 10.34133/bmr.0436

**Published:** 2026-10-21

**Authors:** Ying Qu, Wenyu Zhang, Leilei Song, Xinyu Wang, Xinyi Zhang, Kejian Wu, Zhe Han, Shuai Gao, Huilin Zhao, Cong Song, Runxiao Zheng

**Affiliations:** ^1^ Shandong Provincial Hospital, School of Public Health, Medical Science and Technology Innovation Center, Shandong First Medical University and Shandong Academy of Medical Sciences, Jinan 250117, China.; ^2^ School of Polymer Science and Engineering, Qingdao University of Science and Technology, Qingdao 266042, China.; ^3^ State Key Laboratory of Animal Biotech Breeding, College of Animal Science and Technology, China Agricultural University, Beijing 100193, China.; ^4^ Shandong Medical and Pharmaceutical University, Yantai 264003, China.

## Abstract

Bacterial infections compromise wound healing and represent a growing threat in the era of rising antibiotic resistance. Herein, we developed a multifunctional platform (CN@La@B) that combines bacterial capture, membrane sensitization, visible-light-triggered antibacterial activity, and anti-inflammatory effects. The CN@La@B was prepared by immobilizing dendritic mesoporous lanthanide hydroxide [La(OH)_3_] nanoparticles (La NPs) onto graphite carbon nitride (g-C_3_N_4_) nanosheets to form a topologically structured La(OH)_3_@g-C_3_N_4_ (CN@La) heterostructure, followed by loading of the anti-inflammatory agent berberine. The spiky topological La NPs attached to bacterial surfaces, thereby promoting phospholipid dephosphorylation and enhancing membrane permeability. Under visible-light irradiation, electrons from g-C_3_N_4_ nanosheets were photoexcited to the conduction band and subsequently transferred to La NPs, enabling electron–hole separation and reactive oxygen species generation to eradicate bacteria. Meanwhile, berberine loaded onto the CN@La@B platform helped regulate the wound immune microenvironment. Collectively, both in vitro and in vivo evaluations demonstrated that the CN@La@B killed bacteria and disrupted biofilms, while also modulating inflammation, speeding up wound closure, and supporting tissue regeneration. These findings suggest that CN@La@B is a promising multifunctional platform for infected wound treatment, based on a “flypaper-mimicked” antibacterial strategy that integrates bacterial capture, membrane sensitization, reactive oxygen species generation, and immunomodulation.

## Introduction

Bacterial wound infections arise from bacteria or microbes accumulating within breached skin, provoking intense inflammatory cascades, sepsis, and potentially fatal outcomes, thus constituting a global barrier to tissue repair [1,2]. Globally, bacterial infections now rank as the second leading cause of death, having overtaken both colorectal cancer and AIDS in mortality [3]. Antibiotic administration remains the chief strategy for both preventing and treating bacterial infections [4,5]. Nevertheless, widespread antibiotic use has bred resistance, spawning multidrug-resistant bacteria that now challenge conventional anti-infective therapy [6–8]. Antimicrobial resistance could eclipse cancer as the deadlier threat within 3 decades, claiming an estimated 10 million lives each year by 2050 [9,10]. Therefore, developing non-antibiotic anti-infective strategies is critical to preventing the emergence of bacterial resistance.

Photodynamic antibacterial therapy, recognized for its potent efficacy against infections, carries a markedly lower risk of inducing resistance [11,12]. Two-dimensional nanomaterials have attracted increasing attention due to their large specific surface area, high electron mobility, carrier concentration, and tunable band gap structure [13,14]. Graphite carbon nitride nanosheet (g-C_3_N_4_ NS), as a typical 2-dimensional nanomaterial, has been widely used in the inactivation of bacteria under visible-light irradiation due to its photo-induced reactive oxygen species (ROS) oxidation attack [15,16]. However, photocatalytic activity of g-C_3_N_4_ under visible-light irradiation is hampered by its wide bandgap (2.7 eV) and swift electron–hole recombination [17,18]. The formation of the desirable heterostructure photocatalysts can enhance the transfer of electrons from a narrow bandgap semiconductor to another semiconductor, which is conducive to the separation of carriers and thereby improves the photocatalytic efficiency [19,20]. Moreover, the narrower-bandgap semiconductor should exhibit a conduction-band edge that is energetically elevated relative to the other component [21]. Lanthanide hydroxide (La(OH)_3_), an excellent photocatalyst candidate, can be incorporated into semiconductors to induce the separation of electrons and holes, thereby generating ROS more effectively [22]. Under visible-light irradiation, the electrons of g-C_3_N_4_ can be promoted from the valence band (VB) to the conduction band (CB) and subsequently transfer to La(OH)_3_. This efficient electron–hole spatial separation is conducive to the reaction of free electrons in La(OH)_3_ with oxygen (O_2_) to form superoxide radical anions (O_2_·^−^). Consequently, the g-C_3_N_4_@La(OH)_3_ heterostructure displays markedly stronger antibacterial activity than pristine g-C_3_N_4_ nanoparticles.

In addition, the efficacy of photodynamic antibacterial therapy in promoting wound healing is constrained by several factors, including the extremely short lifetime of ROS (approximately 40 ns), their limited diffusion distance (about 10 nm), and the presence of a pronounced inflammatory response [23–25]. Interestingly, natural surfaces featuring spiky topological structures often exhibit notable adhesive properties. For instance, micrometer-sized pollen grains readily attach to hairy insects for pollination, and nanoscale virus particles display high infectivity toward host cells [26,27]. Inspired by such natural designs, surface roughness has emerged as a key parameter to govern material–bacteria interactions. Notably, nanoscale roughness engineered on titanium and silica surfaces has been shown to substantially enhance bacterial attachment, thereby validating the feasibility of topology-mediated bacterial capture strategies [28,29]. Furthermore, it has been reported that rare earth nanoparticles can transform into sea-urchin-shaped structures on the membrane of gram-negative (G^−^) bacteria, where they induce phospholipid dephosphorylation, thereby increasing membrane fluidity and permeability [30]. To address these limitations, we propose a topological La(OH)_3_@g-C_3_N_4_ heterostructure designed to capture bacteria while continuously depleting membrane phospholipids, thereby enhancing membrane permeability. Consequently, even ROS with a short half-life and limited diffusion distance can effectively eradicate the captured bacteria. Besides, berberine (BBR), an isoquinoline alkaloid, possesses desirable antibacterial, anti-inflammatory, and antioxidant properties for wound healing [31,32].

In this study, we developed a “flypaper-mimicked” strategy that integrates bacterial trapping, membrane sensitization, and ROS generation to achieve efficient wound healing. This platform, denoted as g-C_3_N_4_@La(OH)_3_@BBR (CN@La@B), was engineered by immobilizing dendritic mesoporous La(OH)_3_ nanoparticles (La NPs) onto g-C_3_N_4_ NSs to form a topologically structured La(OH)_3_@g-C_3_N_4_ (CN@La) heterostructure, which subsequently served as the carrier for loading the anti-inflammatory agent BBR. As illustrated in Fig. 1, the spiky topological features of La NPs enable effective bacterial capture through surface adhesive properties. Subsequently, La NPs continuously deplete membrane phospholipids by inducing their dephosphorylation. Under visible-light irradiation, electrons in g-C_3_N_4_ are excited from the VB to the CB and then transferred to La NPs. This efficient spatial separation of electron–hole pairs facilitates the reaction of free electrons accumulated in La NPs with oxygen (O_2_) to generate ^1^O_2_. The as constructed CN@La@B platform exhibited excellent photo-responsive antibacterial activity against *Escherichia coli* even at a low concentration of 50 μg/mL, achieving effective biofilm eradication at infection sites and enabling thorough sterilization. Both in vitro and in vivo assays were integrated to comprehensively evaluate the feasibility of this multifunctional platform for infected wound healing.

**Fig. 1.**
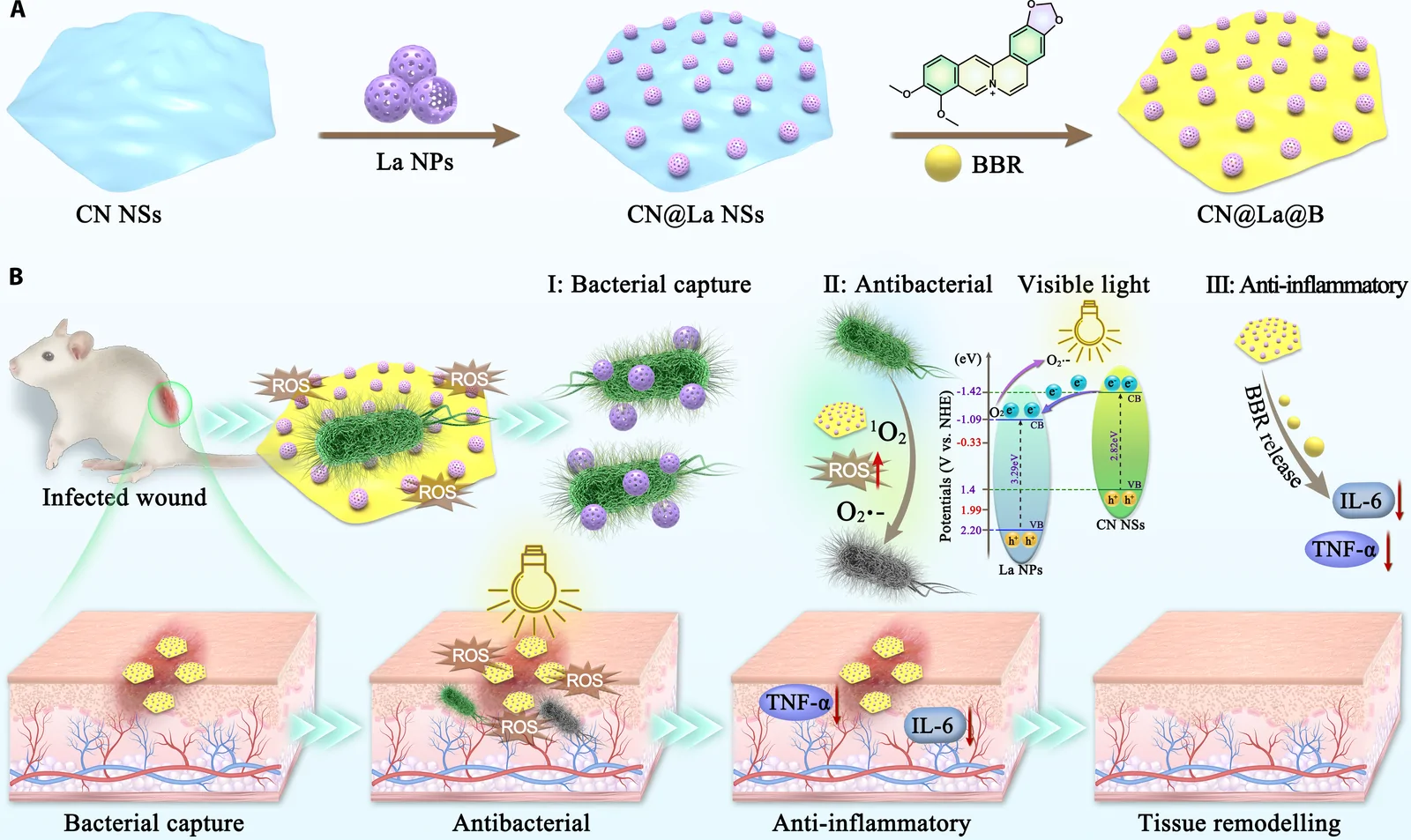
Schematic illustration of the heterostructure platform for bacterial wound healing. (A) Design of CN@La@B as a heterostructure platform. (B) Proposed mechanism of visible-light-enhanced antibacterial and anti-inflammatory effects of CN@La@B for healing bacteria-infected wounds. CN NS, carbon nitride nanosheet; NP, nanoparticle; BBR, berberine; ROS, reactive oxygen species; TNF-α, tumor necrosis factor-alpha; IL-6, interleukin-6; CB, conduction band; VB, valence band; NHE, normal hydrogen electrode.

## Materials and Methods

### Synthesis of CN nanosheets

CN NSs were synthesized according to a previously reported method [33]. Briefly, dicyandiamide (10 g) was heated to 550 °C at a rate of 2 °C/min in a muffle furnace under an air atmosphere for 4 h. The resulting yellow CN bulk powder was then collected after cooling to room temperature. Thereafter, 0.05 g of CN bulk was dispersed in 50 mL of water and subjected to sonication for 24 h. After sonication, the supernatant was collected and centrifuged at a low speed (2,000 rpm, 10 min) to remove unexfoliated CN bulk. The obtained supernatant was regarded as an aqueous dispersion of CN NSs.

### Synthesis of La(OH)_3_ NPs

In brief, citric acid (20.0 mg), hexadecyltrimethylammonium bromide (200.0 mg), sodium salicylate (120.0 mg), hexamethylenetetramine (30 mg), and LaCl_3_·6H_2_O (50 mg) were added to 100 mL of deionized water, followed by the addition of 40 mL cyclohexane. The mixture was vigorously stirred for 1 h and then heated at 70 °C in a water bath for 7 h. The nanoparticles were collected by centrifugation at 12,000 rpm for 30 min and washed 3 times with ethanol. The obtained precipitate was redispersed in 100 mL of ethanol and refluxed for 12 h. The product was collected again by centrifugation to obtain La NPs [34].

### Synthesis of CN@La NSs

La NPs (50 mg) and CN NSs (100 mg) were dispersed in ethanol (20 mL), followed by ultrasonic treatment for 30 min to facilitate the immobilization of La NPs onto the surface of CN NSs. The resulting CN@La NSs were then isolated by centrifugation at 12,000 rpm.

### Synthesis of CN@La@B platform

CN@La NSs (4 mg) were first added into a BBR-containing ethanol solution (1 mg in 20 mL). And then, the suspension was stirred in an ice bath for 12 h to enable BBR incorporation into CN@La NSs. The resulting precipitate was isolated by centrifugation at 5,000 rpm for 5 min and then lyophilized to obtain the CN@La@B platform.

### In vitro release kinetics of BBR from CN@La@B

Firstly, the drug loading efficiency of BBR on CN@La NSs was measured by ultraviolet–visible (UV-vis) spectroscopy using the characteristic absorption peak of BBR at 420 nm. The loading efficiency (LE%) was then calculated according to a previously reported method [35], as follows: LE (%) = (*M*_BBR_ − *C*_BBR_ × *V*_solution_)/*M*_sample_ × 100%, where *M*_BBR_, *C*_BBR_, *V*_solution_, and *M*_sample_ represent the initial amount of BBR, the concentration of BBR in the supernatant after centrifugation (mg/mL), the total solution volume (mL), and the mass of CN NSs (mg), respectively. The in vitro release profile of BBR from CN@La@B was then evaluated using a dialysis method. Briefly, each dialysis bag (molecular weight cutoff: 3,500 Da) containing 2 mL of CN@La@B was immersed in phosphate-buffered saline (PBS) solution (pH = 7.4) at 37 °C. Aliquots of the dialysate were collected at fixed time intervals (0.5, 1, 3, 5, 7, 9, 12, and 24 h) and then replaced with fresh medium of the same volume. Released BBR in the collected dialysate was measured through UV-vis spectroscopy to calculate the cumulative release.

### Investigation of La^3+^ and release efficiency from CN@La@B NPs

To examine the La^3+^ degradation, the 2-mg CN@La@B NPs (at concentrations of CN@La@B NPs) were added to the PBS buffers (2 mL, pH = 7.4) at 37 °C. At certain time intervals, the buffers were collected, and at the same time, 1 mL of fresh buffers was added. The La ion concentrations in the collected buffers were analyzed by inductively coupled plasma mass spectrometry.

### Charge-carrier-transport-behavior analysis

The electronic structure and charge-carrier-transport behavior of the samples were investigated using UV-vis diffuse reflectance spectroscopy (UV-vis DRS) and VB x-ray photoelectron spectroscopy (VB-XPS). UV-vis DRS spectra were recorded to determine the optical absorption properties of La NPs, CN NSs, and CN@La NSs, and the band gap energies were estimated using Tauc plots. VB-XPS measurements were performed to determine the VB maximum (VBM) of samples. The CB minimum (CBM) values were calculated based on the relationship between the band gap energy and the VBM, and the energy band structures were subsequently constructed.

### Measurement of ROS generation

ROS generation by CN@La NSs and CN@La@B under visible-light irradiation was evaluated using multiple probe molecules. Firstly, singlet oxygen (^1^O_2_) production was assessed using 9,10-anthracenediyl-*bis*(methylene)dimalonic acid (ABDA) and 1,3-diphenylisobenzofuran (DPBF) as specific probes. Changes in absorbance of ABDA (380 nm) and DPBF (415 nm) were monitored by UV-vis spectroscopy. Notably, control experiments were performed under dark conditions for comparison.

### Phosphate-adsorption study

To assess phosphate adsorption, CN@La@B was incubated in a phosphate-containing solution. After adsorption, the samples were collected, washed, and dried for further characterization. XPS was then carried out to examine the chemical states and elemental composition of CN@La@B before and after phosphate adsorption. Both survey and high-resolution spectra, including La 3d, O 1s, P 2p, and C 1s, were recorded and analyzed.

### Bacterial capture evaluation

Bacterial suspensions were dispersed in 1 mL of saline containing CN@La@B (100 μg/mL) and incubated for 30 min. After incubation, the treated bacterial suspensions were fixed with 2.5% glutaraldehyde overnight. The samples were subsequently dehydrated in graded ethanol solutions and examined by scanning electron microscopy to assess bacterial capture and aggregation. For quantitative determination of bacterial capture efficiency, *E. coli* suspensions [1 × 10^7^ colony-forming units (CFU)/mL] were divided into 6 groups: PBS, CN NSs, La_2_O_3_ NPs, La NPs, CN@La NSs, and CN@La@B. All NP groups were adjusted to a final concentration of 100 μg/mL. All samples were incubated at 37 °C for 30 min under gentle shaking (150 rpm). Afterwards, the mixtures were allowed to stand for 5 min to facilitate sedimentation of nanoparticle–bacteria aggregates. The supernatants containing uncaptured free bacteria were carefully collected. Serial dilution was performed, and 100 μL of each diluted suspension was spread onto Luria–Bertani (LB) agar plates.

### Bacterial membrane permeability

The outer-membrane permeability of *E. coli* strains was evaluated using *N*-phenyl-1-naphthylamine (NPN) as a fluorescent probe, following the method of Dubey et al. [36] with minor modifications. Bacterial suspensions (1 × 10^6^ CFU/mL) in PBS were treated with PBS, CN NSs, La_2_O_3_ NPs, La NPs CN@La NSs, or CN@La@B for 1 h and then mixed with 200 μM NPN to a final volume of 300 μL. Fluorescence was recorded at *λ*_ex_ = 350 nm and *λ*_em_ = 420 nm using a FluoStar Omega plate reader (BMG Labtech, Germany). An increase in fluorescence intensity indicated enhanced outer-membrane permeability [37].

### Antibacterial activity evaluation

Plate counting, growth-curve analysis, and live/dead bacterial staining assays were used to evaluate the antibacterial activity of CN@La@B. In the plate counting assay, *E. coli* suspensions (1 × 10^7^ CFU/mL, 100 μL) were incubated with PBS, CN NSs, CN@La NSs, and CN@La@B (50 μg/mL) in centrifuge tubes for 6 h at 37 °C. The suspensions were then serially diluted with sterile PBS, and each dilution (30 μL) was spread on agar plates followed by incubation at 37 °C for 12 h. After counting CFU, bacterial survival rate was calculated: survival rate (%) = *A*_E_/*A*_C_ × 100%, where *A*_C_ represents bacterial concentrations of the control and *A*_E_ represents bacterial concentrations of experimental groups. For growth-curve analysis, *E. coli* suspensions (1 × 10^7^ CFU/mL) were incubated with PBS, CN NSs, CN@La NSs, and CN@La@B (50 μg/mL) under dark conditions or visible light for 30 min, followed by transfer into LB broth (10 mL) and incubation with shaking at 200 rpm at 37 °C. Bacterial growth was monitored by measuring the optical density at 600 nm (OD_600_) at designated time points. For live/dead staining, *E. coli* suspensions (1 × 10^7^ CFU/mL) were incubated with PBS, CN NSs, CN@La NSs, and CN@La@B (50 μg/mL) for 4 h under visible-light or dark conditions, then stained with acridine orange/propidium iodide dye and observed.

### Evaluation of anti-inflammatory activity in LPS-stimulated RAW264.7 cells

To induce an inflammatory response, RAW264.7 macrophages were treated with lipopolysaccharide (LPS, 1 μg/mL) for 6 h. Following LPS stimulation, RAW264.7 cells were incubated with PBS, CN NSs, CN@La NSs, and CN@La@B (50 μg/mL) for 24 h. Subsequently, the culture supernatants were collected, and the protein levels of interleukin-6 (IL-6) and tumor necrosis factor-α (TNF-α) were measured using enzyme-linked immunosorbent assay kits according to the manufacturer’s instructions. The results were compared between the treated and untreated groups.

### In vivo infected wound model establishment

Female BALB/c mice (6 to 8 wk old, 18 to 19 g) were purchased from Pengyue Laboratory Animal Technology Co., Ltd. (Shandong, China) and acclimatized for 1 week before the experiments. All experimental procedures were approved by the Animal Care and Use Committee of Shandong First Medical University (No. W202512290947) and followed China’s national laboratory animal welfare regulations and institutional ethical guidelines. After anesthesia, the dorsal fur was shaved using a surgical razor, and the skin was disinfected with 75% ethanol. Full-thickness wounds (10 mm in diameter) were created on the dorsal region of each mouse using a sterile biopsy punch. To induce infection, wounds were inoculated with 50 μL of *E. coli* suspension (1 × 10^8^ CFU/mL), followed by a 30-min attachment period.

### In vivo evaluation of wound healing efficacy

After successful establishment of the wound model, the animals were randomly assigned to 5 experimental groups: negative control (PBS), PBS + Light, CN NSs, CN@La NSs, CN@La@B, and CN@La@B under visible-light irradiation (CN@La@B + Light). The corresponding treatments were applied to the wound sites, and the dressings were replaced every 48 h. Among them, visible-light irradiation refers to exposing the wound area to visible light. The CEL-HXF300-T3 xenon lamp (Ceaulight, Beijing, China) was used as the excitation light and was applied for 30 min, a 300-W Xe lamp was used to simulate sunlight illumination on days, and the distance between the sample and the lamp was set to 12 cm, with a 0.2 W/cm^2^ and 420- to 780-nm irradiation wavelength range [11,18].

After topical administration of different formulations, visible-light irradiation was performed on assigned groups at scheduled time points (days 0, 3, 5, 7, and 14). Wound-surface temperature was continuously recorded using an infrared thermal imager throughout each irradiation procedure. Guided by the visible wound boundary, thermal images were processed to obtain the peak temperature over the wound bed using thermal analysis software [38]. The PBS + Light group was established as a light-only control to assess temperature alterations triggered by light irradiation perse. For wound-closure evaluation, digital photographs of wounds were captured on days 0, 3, 5, 7, and 14 posttreatment to track wound contraction. Wound area was quantified using ImageJ software (NIH, USA), and the relative residual wound area was further computed. To evaluate antibacterial activity in vivo, wound exudate samples were harvested on day 3 posttreatment for bacterial quantification by CFU assay. On day 14, wound tissues along with the surrounding skin were harvested after anesthesia. Samples were immersed in 4% paraformaldehyde for 24 h to complete fixation. After gradient ethanol dehydration, the specimens were embedded in paraffin and cut into continuous sections with a thickness of 5 μm. Hematoxylin–eosin (H&E) and Masson’s trichrome staining were performed on the sliced samples to observe tissue regeneration status and evaluate collagen deposition levels.

For immunofluorescence analysis, additional sections on day 7 posttreatment were stained with antibodies against TNF-α, IL-6, CD31, and vascular endothelial growth factor (VEGF) to evaluate inflammatory response and angiogenesis.

### Statistical analysis

Quantitative data are presented as mean ± standard deviation. The numerical comparison was performed using one-way analysis of variance followed by Tukey post hoc comparison. Statistical significance was defined as *P < 0.05, **P < 0.01, ***P < 0.001, and ****P < 0.0001.

## Results and Discussion

### Synthesis and characterization of the lanthanide heterostructure platform

In this study, a lanthanide heterostructure platform, named CN@La@B, was synthesized by immobilizing dendritic mesoporous La NPs onto CN NSs and subsequently loading BBR (Fig. 1A). Transmission electron microscopy (TEM) analyses exposed that the exfoliated CN NSs exhibited a smooth and ultrathin sheet-like morphology without apparent pores (Fig. 2A), indicating the effective delamination of CN bulk. In contrast, the synthesized La NPs displayed a uniform spherical morphology with dendritic mesostructures (Fig. 2B). After hybridization, the CN@La NSs exhibited a substantially roughened surface (Fig. 2C), where La NPs were homogeneously anchored onto the CN NSs without observable aggregation. This suggests strong interfacial interaction and improved dispersion of La NPs, which facilitates the exposure of a greater number of active sites. Upon further loading of BBR, the CN@La@B showed enhanced contrast in TEM images (Fig. 2D), indicating successful incorporation of BBR molecules. The CN NSs, La NPs, CN@La NSs, and CN@La@B NSs exhibited TEM-derived average geometric diameters of 388 ± 125.1 nm, 217 ± 78.4 nm, 507 ± 62.9 nm, and 512 ± 79.8 nm, respectively (Fig. S1). Subsequently, the crystal structures of CN bulk, CN NSs, La NPs, and CN@La NSs were characterized via x-ray diffraction (XRD; Fig. 2E). Compared with the standard XRD card (JCPDS No. 87-1526) [39,40], CN bulk displayed 2 characteristic diffraction peaks at 2*θ* = 13.0° and 27.4°, attributable to the (100) and (002) planes, respectively. After ultrasonic exfoliation, the disappearance of the (100) peak indicated the disruption of in-plane structural ordering in CN NSs. Meanwhile, the amorphous nature of La NPs and the unchanged diffraction pattern of CN@La NSs demonstrated that the structural integrity of CN was preserved during hybridization. The interplanar spacing (*d*_hkl_) can be calculated as follows:

$$d_{\mathrm{hkl}}=\frac{\lambda }{2\;\text{sin}\;\theta }$$ (1)

where *λ* = 0.15406 nm is the wavelength of Cu-Kα x-ray radiation and *θ* represents the diffraction angle. The calculated *d*_(100)_ and *d*_(002)_ values of CN bulk were 0.680 nm and 0.325 nm, respectively. After ultrasonic exfoliation, the disappearance of the (100) peak indicated the disruption of in-plane structural ordering in CN NSs. Meanwhile, the (002) diffraction peak slightly shifted to a higher 2*θ* value of 27.6°, corresponding to a reduced interplanar spacing of 0.323 nm, revealing weakened interlayer stacking interaction after exfoliation. For CN@La NSs, the position of the (002) peak remained at 27.6° with an identical *d*_(002)_ spacing of 0.323 nm. The successful fabrication of the CN@La NSs was further verified via Fourier-transform infrared spectroscopy (Fig. 2F). For pure CN NSs, multiple characteristic absorption peaks between 1,200 and 1,700 cm^−1^ were ascribed to the stretching and bending modes of C=N and C–N bonds in the triazine ring framework. Additionally, broad bands located around 3,100 cm^−1^ and 3,400 cm^−1^ were assigned to the stretching vibrations of N–H and O–H bonds, respectively. After hybridization, a new absorption peak appeared at around 602 cm^−1^ in the CN@La NSs, which is attributed to the stretching vibration of La–O bonds. These results confirm the anchoring of La NPs onto CN NSs.

**Fig. 2.**
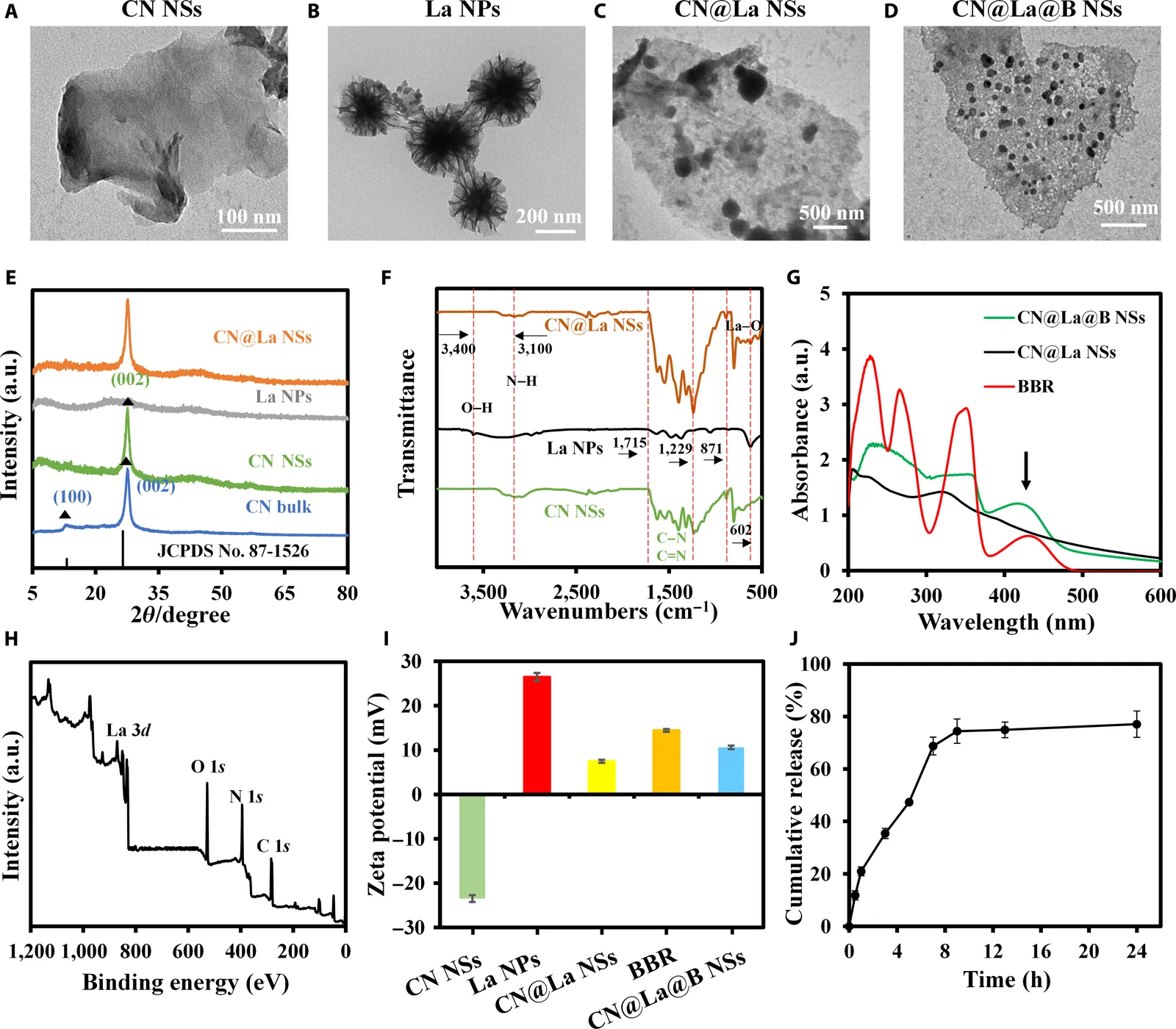
Characterization of CN@La@B and intermediate products. Transmission electron microscopy images of (A) CN NSs, (B) La NPs, (C) CN@La NSs, and (D) CN@La@B platforms. (E) X-ray diffraction patterns of CN bulk, CN NSs, La NPs, and CN@La NSs. JCPDS, Joint Committee on Powder Diffraction Standards. (F) Fourier-transform infrared spectroscopy and (G) UV-vis spectra of CN NSs, La NPs, and CN@La NSs. (H) X-ray photoelectron spectroscopy (XPS) survey spectrum of CN@La@B. (I) Zeta potential of CN NSs, La NPs, CN@La NSs, BBR, and CN@La@B (*n* = 3). (J) Release profiles of BBR from CN@La@B (*n* = 3).

Next, the successful loading of BBR onto CN@La NSs was verified by UV-vis spectroscopy. As shown in Fig. 2G, CN@La@B exhibited a characteristic absorption peak at ~ 420 nm, corresponding to BBR, confirming its successful incorporation. XPS analysis was further performed to investigate the elemental composition of CN@La@B (Fig. 2H). La, O, C, and N were clearly detected in the survey spectrum, matching the composition of the hybrid system. Meanwhile, the surface charge evolution during the assembly process was evaluated by zeta-potential measurements (Fig. 2I). The zeta potential increased from −23.5 ± 0.8 mV for CN NSs to 7.5 ± 0.3 mV for CN@La NSs, indicating the successful loading of positively charged La species. After BBR loading, the zeta potential further increased to 10.6 ± 0.4 mV, which can be attributed to the cationic nature of BBR molecules. The loading capacity of BBR was quantified by UV-vis absorption spectroscopy based on a calibration curve (Fig. S2), yielding a value of 30.3 ± 0.2 μg per 100 μg of CN NSs. In addition, the drug release behavior of CN@La@B was evaluated under physiological conditions (PBS, pH = 7.4, and 37 °C). As shown in Fig. 2J, within the initial 8 h, BBR exhibited a rapid release, followed by a sustained release phase. The cumulative release reached 77.1% at 24 h. The lanthanum-ion release behavior of CN@La@B NPs was investigated in PBS (pH 7.4) at 37 °C using inductively coupled plasma mass spectrometry to quantify cumulative La^3+^ concentrations over time. As shown in Fig. S3, a rapid initial release of La^3+^ was observed within the first 60 min, followed by a sustained and slow-increase phase. The release rate gradually slowed down after 120 min and tended to plateau at approximately 180 min, indicating that the La^3+^ release reached a near-saturated state. Such time-dependent La^3+^ release profile suggests that CN@La@B NPs can continuously liberate La^3+^ under physiological conditions. The released La^3+^ may participate in perturbing bacterial membrane homeostasis and partially contribute to the overall antibacterial performance of the nanoplatform. Taken together, these results confirm the successful fabrication of CN@La@B with efficient BBR loading and controlled release capability.

### Band structure and charge transfer mechanism

In order to elucidate the intrinsic photoelectronic properties of heterostructure systems, La NPs, CN NSs, and CN@La NSs were selected as the representative models to investigate the band structure and charge transfer behavior. As can be seen in Fig. 3A to C, UV-vis DRS analysis revealed that the band gap energies of La NPs, CN NSs, and CN@La NSs were 3.29 eV, 2.82 eV, and 2.44 eV, respectively, indicating enhanced visible-light absorption after heterojunction formation. The VB positions were determined by VB-XPS, and the corresponding CBM values were calculated. From the VB-XPS spectra (Fig. 3D to F), the VBM values of La NPs, CN NSs, and CN@La NSs were determined to be 2.2 eV, 1.4 eV, and 1.3 eV, respectively. The derived CBM values were −1.09 eV for La NPs, −1.42 eV for CN NSs, and −1.14 eV for CN@La NSs, enabling the construction of their respective energy band structure diagrams. Based on these results, a typical heterostructure was established between CN NSs and La NPs. Such a heterostructure system effectively promotes the spatial separation of charge carriers while maintaining strong redox ability. As illustrated in Fig. 3G, under visible-light irradiation, the electrons of CN NSs are excited from the VB to the CB and then migrate to La NPs, and the efficient spatial separation of electrons and holes enables free electrons on La NPs to react with O_2_ to generate O_2_^·−^ and ^1^O_2_. These characteristics enable efficient charge carrier separation in the CN@La NS heterostructure, leading to enhanced visible-light-induced ROS generation.

**Fig. 3.**
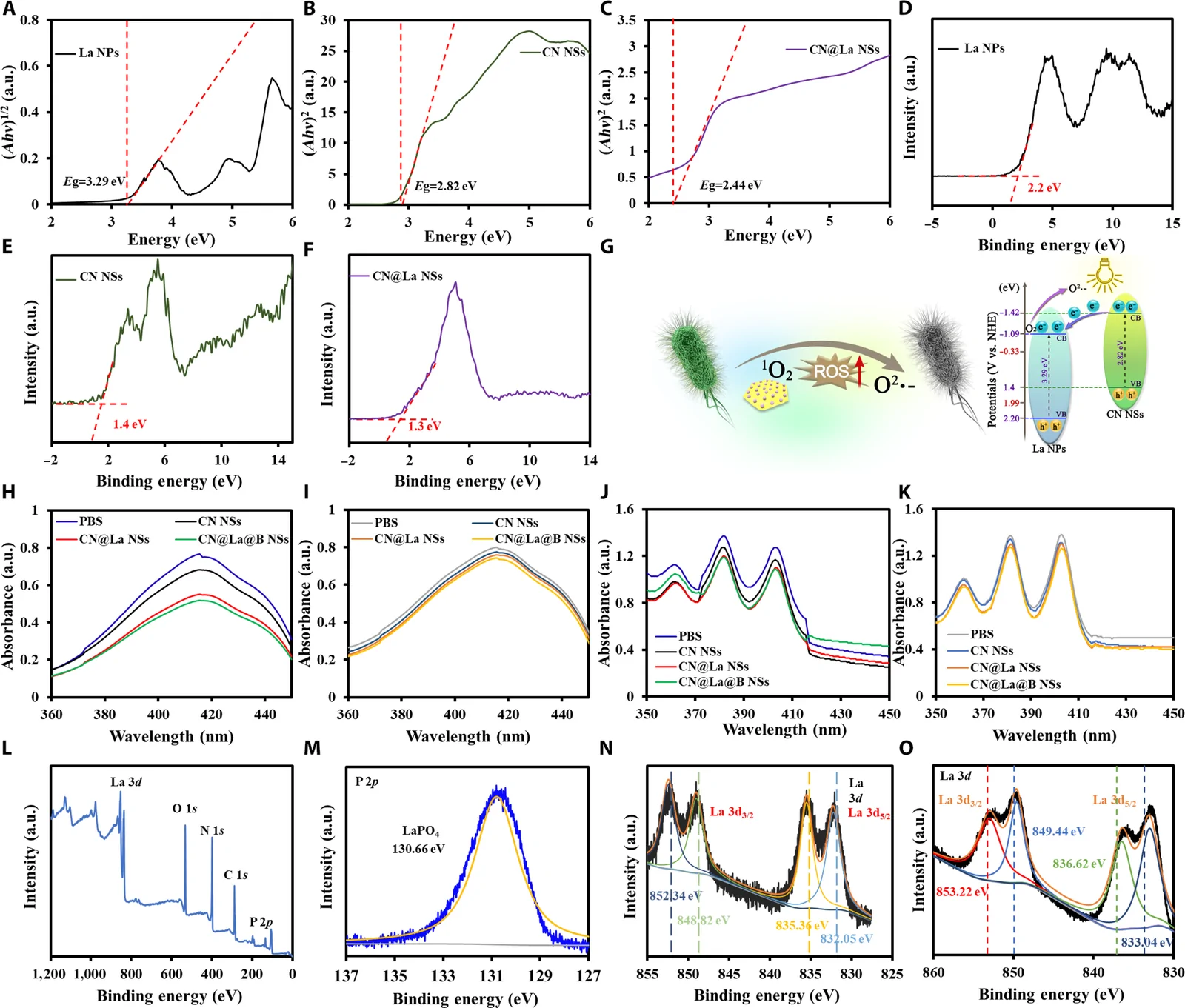
Charge transfer mechanism, ROS generation performance, and phosphate adsorption of CN@La@B. Tauc plots derived from UV-vis diffuse reflectance spectra of (A) La NPs, (B) CN NSs, and (C) CN@La NSs. Valence band XPS spectra of (D) La NPs, (E) CN NSs, and (F) CN@La NSs. (G) Schematic illustration of the charge transfer pathway and the activation mechanisms of O_2_ for ROS generation. UV-vis absorption spectra of 1,3-diphenylisobenzofuran in phosphate-buffered saline (PBS), CN NSs, CN@La NSs, and CN@La@B solutions (equivalent CN NS content: 50 μg/mL) under (H) visible-light irradiation and (I) dark conditions. UV-vis absorption spectra of 9,10-anthracenediyl-*bis*(methylene)dimalonic acid in PBS, CN NSs, CN@La NSs, and CN@La@B solutions (equivalent CN NS content: 50 μg/mL) under (J) visible-light irradiation and (K) dark conditions. (L) XPS survey spectrum and (M) high-resolution P 2p spectrum of CN@La@B after phosphate adsorption. High-resolution XPS La 3d spectrum of (N) pristine CN@La@B and (O) CN@La@B after phosphate adsorption.

### ROS-generation performance

The ROS-generation performance of both CN@La NSs and CN@La@B was further evaluated to verify their photocatalytic activity. Firstly, the ^1^O_2_ generation capacity of CN@La@B was assessed using DPBF and ABDA as probes. The characteristic absorbance of DPBF at 415 nm declined when incubated with CN@La NSs or CN@La@B under visible-light irradiation (Fig. 3H). In sharp contrast, only a slight decrease in absorbance of DPBF was observed under dark conditions (Fig. 3I). Similarly, as shown in Fig. 3J, at an equivalent CN NS concentration of 50 μg/mL, the absorbance of ABDA decreased in CN@La NSs and CN@La@B under visible-light irradiation, indicating efficient generation of ^1^O_2_. In contrast, no obvious change in the characteristic UV-vis absorption peaks of ABDA was observed under dark conditions (Fig. 3K). Both results consistently confirmed efficient ^1^O_2_ generation.

### Phosphate adsorption and membrane disruption mechanism

Given the high affinity of La NPs for phospholipid phosphate head groups, which promotes membrane disruption and phospholipid dephosphorylation [41], the CN@La@B platform was used to probe the phosphate-adsorption behavior and the underlying antibacterial mechanism. XPS was employed to examine chemical states and elemental composition of CN@La@B before and after phosphate adsorption. Figure S4 shows that the high-resolution N 1s spectrum of pristine CN@La@B can be deconvoluted into 4 characteristic peaks of the g-C_3_N_4_ framework, corresponding to tertiary nitrogen (N-(C)_3_, 396.94 eV), sp^2^-hybridized nitrogen (C=N-C, 395.3 eV), uncondensed amino groups (397.96 eV), and π-excitation (403.24 eV), confirming the structural integrity of CN prior to adsorption. In contrast to the XPS survey spectrum of pristine CN@La@B (Fig. 2H), the spectrum of CN@La@B after phosphate adsorption exhibited a new peak at ~134 eV (Fig. 3L), which is characteristic of P 2p, confirming the successful uptake of phosphate by CN@La@B. Further analysis of the high-resolution P 2p spectrum revealed a typical peak at 130.66 eV, suggesting the formation of LaPO_4_ species on the surface of CN@La@B (Fig. 3M) [42]. For the La 3d spectrum of pristine CN@La@B, 2 pairs of characteristic peaks assigning to La 3d_5/2_ and La 3d_3/2_ were observed at 832.05/835.36 eV and 848.82/852.34 eV, respectively (Fig. 3N). After phosphate adsorption, both La 3d_5/2_ and La 3d_3/2_ peaks shifted to higher binding energies (833.04/836.62 eV and 849.44/853.22 eV, respectively) (Fig. 3O). This positive shift indicates electron transfer from La sites and supports the formation of La–O–P coordination structures [43,44]. In addition, compared with pristine CN@La@B, the O 1s spectrum after phosphate adsorption can be deconvoluted into 3 distinct peaks at 528.11, 528.97, and 529.63 eV, corresponding to lattice oxygen (La–O), –OH, and H_2_O, respectively (Fig. S5). Notably, after adsorption, the content of surface –OH decreased from 46.4% to 37.6%, indicating a ligand exchange process between hydroxyl groups and phosphate ions, leading to the formation of inner-sphere complexes. Furthermore, the high-resolution C 1s spectrum showed 2 main peaks at 283.1 and 284.71 eV, belonging to C=O and O–C=O groups, respectively. After phosphate adsorption, these peaks exhibited slight red shifts, accompanied by an increase in the relative content of O–C=O groups from 43.6% to 46.8% (Fig. S6). This change suggests that surface carbonate species (CO_3_^2−^) were partially replaced by phosphate ions, further contributing to phosphate adsorption [45]. Thus, a synergistic antibacterial mechanism is proposed for CN@La@B. Under visible-light irradiation, CN@La@B facilitates charge separation and generates ROS, which induce oxidative damage to bacterial cells. Simultaneously, the La-based components selectively capture phosphate ions at the nano-bio interface, leading to dephosphorylation of membrane phospholipids and disruption of membrane structure. This non-ROS pathway further enhances antibacterial activity. The combination of ROS-mediated oxidative stress and phosphate-induced membrane disruption results in a dual antibacterial mechanism with substantially enhanced efficacy. Such a synergistic effect not only improves antibacterial performance but also provides a robust foundation for subsequent anti-inflammatory activity and accelerated wound healing.

### Bacterial targeting and antibacterial performance in vitro

Inspired by the favorable band structure, efficient ROS generation capability, and phosphate-adsorption-induced membrane disruption mechanism demonstrated above, the bacterial targeting ability and antibacterial performance of CN@La@B were further evaluated in vitro. To evaluate the contribution of surface topology and distinguish it from electrostatic effects, we synthesized smooth spherical La_2_O_3_ NPs as a morphology control sample. The prepared La_2_O_3_ NPs possess a uniform particle size of approximately 50 nm and a zeta potential of +49 mV, which is comparable to that of dendritic La NPs (Fig. S7A to C). As observed in Fig. 4A and Fig. S8, the *E. coli* surface in the CN NS and La_2_O_3_ NP groups remained smooth and intact, with no obvious nanoparticle adhesion. In contrast, a large number of CN@La NSs and CN@La@B (indicated by red arrows) were observed to adhere to the bacterial surface. This enhanced adhesion can be attributed to the structural characteristics of bacterial cell surfaces, which are typically enriched with filamentous pili (several micrometers in length and 5 to 8 nm in diameter), facilitating nanoparticle attachment [46]. To further quantify the bacterial trapping capability of these nanoplatforms, a supernatant-based CFU counting assay was conducted to calculate bacterial capture efficiency (Figs. S9 and S10). The PBS and La_2_O_3_ groups exhibited low capture efficiencies. Compared with the CN NS group, markedly enhanced bacterial capture performance was observed for the CN@La NS group. CN@La@B also showed high bacterial capture efficiency, supporting an important contribution of its spiky surface topology to bacterial adhesion. Notably, comparable capture efficiencies were obtained for CN@La@B and CN@La@B + Light, indicating that bacterial capture was largely independent of light irradiation. Subsequently, the antibacterial activity of different NSs was evaluated. As depicted in Fig. 4B and Fig. S11, CFU assays revealed that CN@La NSs and CN@La@B exhibited moderate antibacterial activity under dark conditions, which can be attributed to phosphate-adsorption-induced membrane disruption. In contrast, under visible-light irradiation, both CN@La NSs and CN@La@B exhibited significant antibacterial activity, with bacterial clearance rates of 86.6% and 93.4%, respectively.

**Fig. 4.**
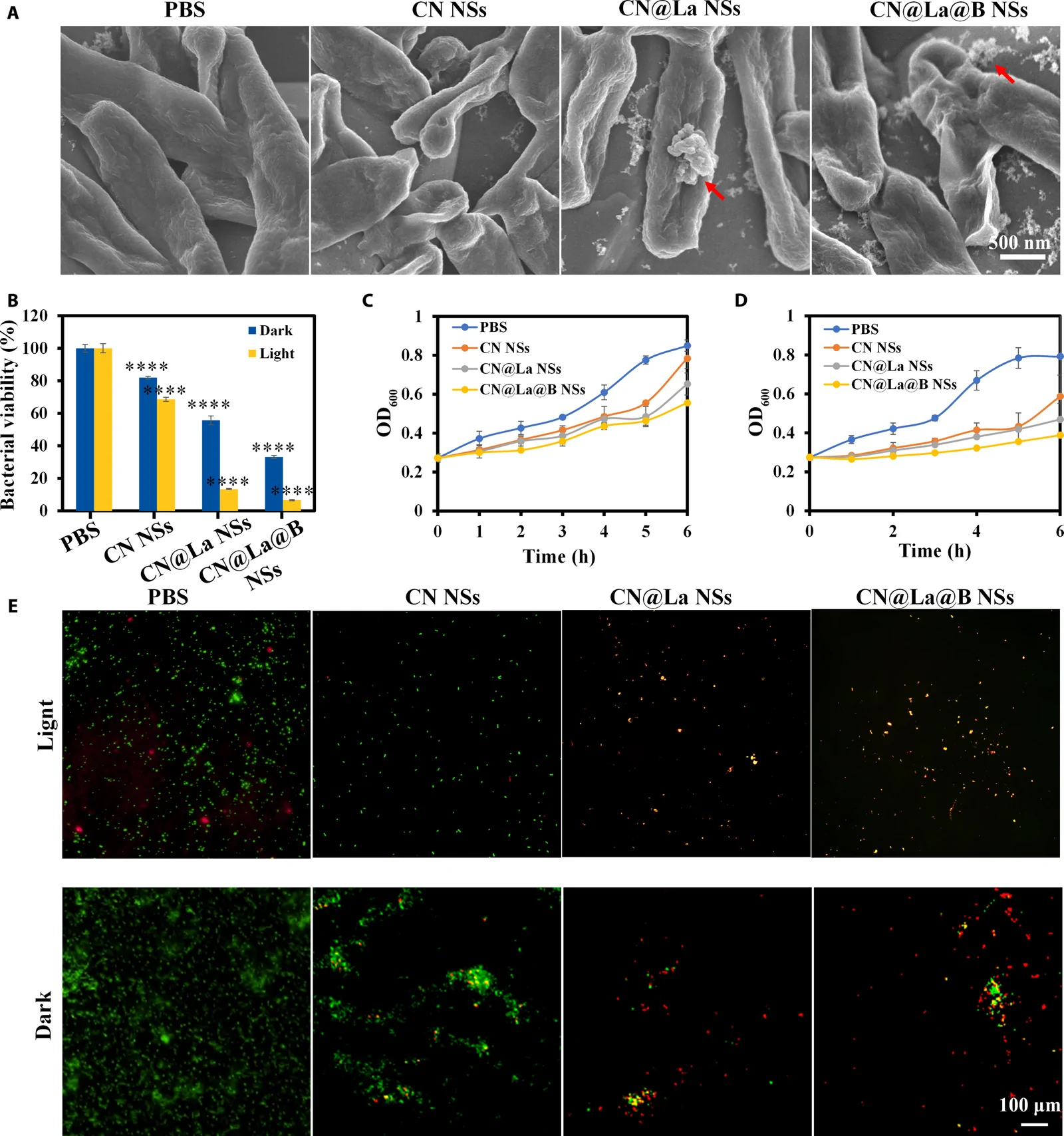
Bacterial capture and antibacterial performance of CN@La@B. (A) Scanning electron microscopy images of *Escherichia coli* after various treatments (scale bar: 500 nm). (B) Bar chart corresponding to CFU counts of *E. coli* colonies on Luria–Bertani agar plates after various treatments (*n* = 5). ****P < 0.0001. Growth curves of *E. coli* after different treatments for more than 6 h under (C) visible-light irradiation and (D) dark conditions (*n* = 5). (E) Fluorescence images of *E. coli* stained with propidium iodide (red, dead bacteria) and acridine orange (green, live bacteria) after various treatments (scale bar: 100 μm).

To further validate the antibacterial performance, bacterial proliferation was tested by monitoring the optical density at 600 nm (OD_600_) (Fig. 4C and D). Under dark conditions, the OD_600_ values of *E. coli* increased from approximately 0.27 to 0.56, 0.65, 0.78, and 0.84 after 6-h treatment with PBS, CN, CN@La, and CN@La@B, respectively, indicating continuous bacterial proliferation. In contrast, under visible-light irradiation, the OD_600_ values in the CN@La and CN@La@B groups remained at low levels, suggesting effective inhibition of bacterial growth. These results indicate that while NSs alone exhibit limited antibacterial activity, their combination with visible light substantially enhances antibacterial efficacy. In addition, live/dead bacterial staining using acridine orange and propidium iodide was further performed to visualize bacterial viability. As seen in Fig. 4E, live bacteria emitted green fluorescence, while dead bacteria exhibited red fluorescence. As expected, bacteria in the PBS group showed predominantly green fluorescence, meaning the bacteria survive. In comparison, bacteria in CN@La NS and CN@La@B groups under visible-light irradiation displayed strong red fluorescence, indicating extensive bacterial death. Notably, partial red fluorescence was also observed in the absence of visible light, suggesting that phosphate-adsorption-induced membrane disruption may contribute to antibacterial activity even without photoactivation. Overall, these data demonstrate that CN@La@B displays enhanced bacterial targeting ability and potent antibacterial activity through the synergistic effects of light-triggered ROS generation and phosphate-induced membrane disruption. To explore whether the antibacterial action of CN@La@B NSs was associated with outer-membrane damage, the outer-membrane permeability of *E. coli* was determined using NPN as a fluorescent probe. NPN is a hydrophobic fluorescent dye that exhibits augmented fluorescence upon penetrating compromised bacterial outer membranes; higher fluorescence intensity corresponds to greater outer-membrane permeabilization. As displayed in Fig. S12, the PBS and PBS + Light groups yielded low fluorescence signals, suggesting that visible-light irradiation alone scarcely disrupted bacterial outer-membrane integrity. Treatment with CN NSs produced a moderate increase in fluorescence intensity, and further elevation was observed for the CN@La NS group. Substantially stronger fluorescence was detected after incubation with CN@La@B NSs. Importantly, the CN@La@B NSs + Light group generated the maximum fluorescence value among all tested groups. These findings revealed that CN@La@B NSs could perturb the bacterial outer membrane, and such membrane-damaging capability was markedly amplified under visible-light irradiation. The aggravated outer-membrane permeabilization contributes to the potent bactericidal effect of the nanoplatform.

### Cytocompatibility and anti-inflammatory activity in vitro

As wound dressings are in direct contact with biological tissues, their cytocompatibility must be considered before biomedical applications. Therefore, the cytotoxicity of CN NSs, CN@La NSs, and CN@La@B at different concentrations was evaluated using L929 fibroblasts. As presented in Fig. S13, cell viability remained above 80% after incubation with the tested samples at a concentration of 100 μg/mL, indicating acceptable cytocompatibility within this concentration range. Even at 200 μg/mL, the CN@La@B group retained a cell viability of 73.3%. Notably, the slight decrease in viability at elevated concentrations may be attributed to the generation of ROS under visible-light irradiation, which can affect cellular metabolic activity. Overall, these results suggest that CN@La@B has favorable cytocompatibility and is suitable for subsequent wound healing applications.

Considering the well-documented anti-inflammatory activity of BBR [26], the anti-inflammatory potential of CN@La@B was further examined using LPS-stimulated RAW264.7 macrophages. Firstly, the cytotoxicity of CN@La@B toward RAW264.7 cells was tested. The cell viability remained above 80% after treatment with the tested samples even at a concentration of 100 μg/mL (Fig. S14), which is suitable for subsequent experiments. Next, the expression levels of pro-inflammatory cytokines were analyzed under LPS-induced inflammatory conditions (1 μg/mL). Figures S15 and S16 show that LPS stimulation up-regulated the expression of IL-6 and TNF-α. By comparison, treatment with CN@La@B suppressed the expression of both IL-6 and TNF-α. Notably, CN@La@B exhibited a stronger inhibitory effect than CN@La NSs, indicating the key role of BBR in modulating inflammatory responses. These data prove that CN@La@B attenuates macrophage-mediated inflammatory responses, thereby providing additional therapeutic benefits for wound healing.

### In vivo therapeutic efficacy of CN@La@B for infected wounds

The in vivo therapeutic efficacy of CN@La@B in infected wounds was further explored. First of all, a full-thickness *E. coli*-infected wound model was built in mice, followed by different treatments (Fig. 5A). The animals were divided into 6 groups: PBS, PBS + Light, CN NSs, CN@La NSs, CN@La@B, and CN@La@B plus under visible-light irradiation (CN@La@B + Light). The CEL-HXF300-T3 xenon lamp (Ceaulight) served as the excitation light source. The sample-to-lamp distance was fixed at 12 cm, delivering irradiation at 0.2 W/cm^2^ over the 420- to 780-nm visible-light range. Relevant therapeutic treatments were performed every 48 h. During phototherapy, negligible temperature variation was observed for the tested materials. The wound-bed temperature fluctuated merely by 0.1 to 0.5 °C (Figs. S17 and S18). It is possible to exclude thermal effects as the cause of phototherapy’s therapeutic outcomes. Such negligible temperature elevation cannot induce thermal-mediated bactericidal effects, confirming that the antibacterial performance originates from photocatalytically generated ROS rather than photothermal heating. Also, the progression of wound healing was monitored by digital imaging on days 0, 3, 5, 7, and 14. As shown in Fig. 5B and C, all treatment groups exhibited faster wound closure than the PBS, PBS + Light group based on quantitative wound area analysis. The CN@La@B + Light group exhibited the fastest healing rate, reaching 82.7% on day 7, compared with 78.4% in the CN@La@B group without light irradiation. On day 14, the CN@La@B + Light group achieved nearly complete wound closure (98.3%), whereas the PBS group showed a lower healing rate (51.7%).

**Fig. 5.**
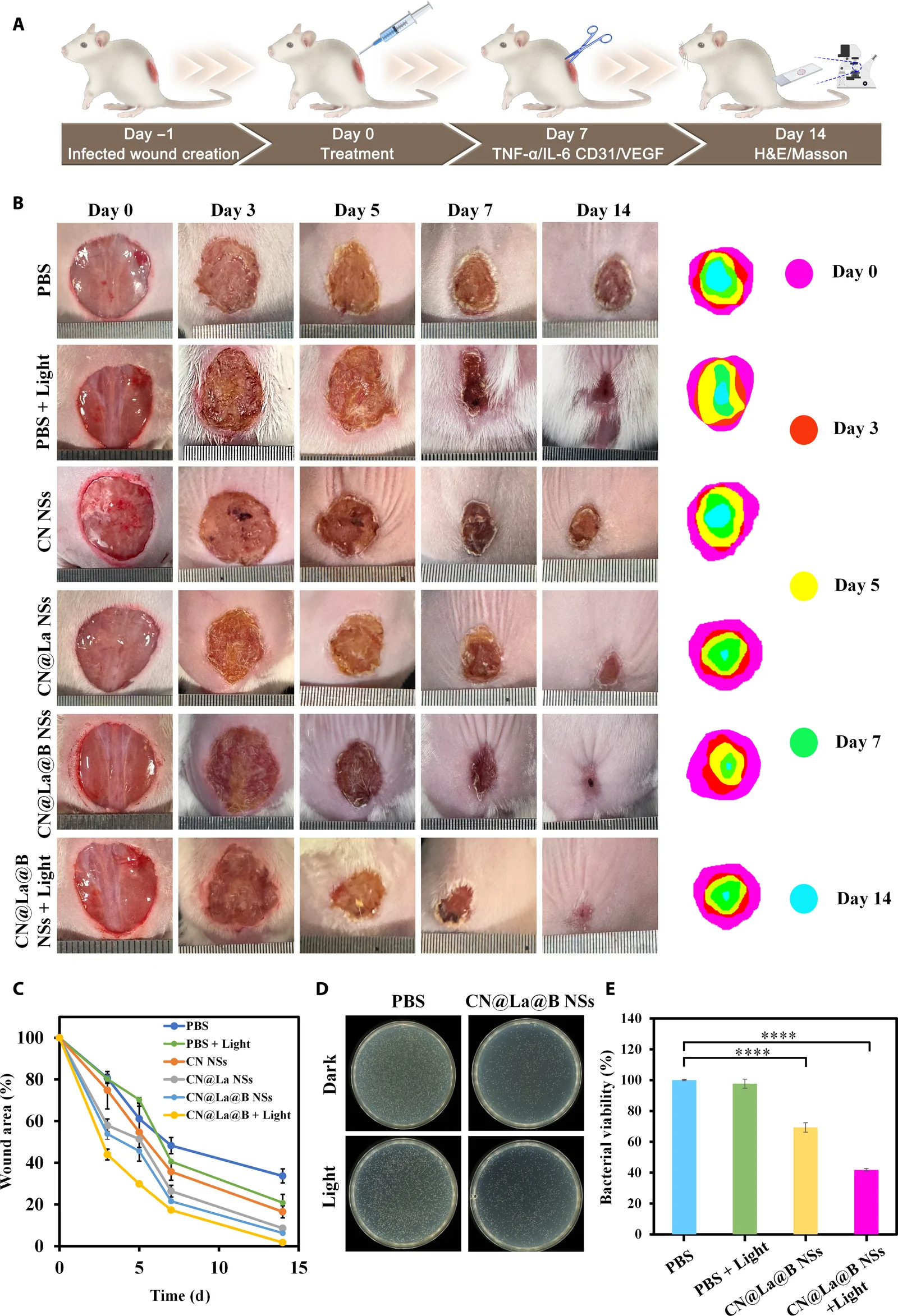
In vivo therapeutic efficacy of CN@La@B for infected wounds. (A) Schematic illustration of the experimental procedure for treating *E. coli*-infected wounds in mice. VEGF, vascular endothelial growth factor; H&E, hematoxylin–eosin. (B) Photographs and (C) quantitative analysis of infected wounds at days 0, 3, 5, 7, and 14 following various treatments (*n* = 5). (D) Representative images and (E) corresponding statistical analysis of bacterial colonies under different treatment conditions (*n* = 5). ****P < 0.0001.

To assess antibacterial efficacy in vivo, wound exudates were harvested on day 3, then cultured on agar plates and subjected to CFU counting. As can be seen in Fig. 5D and E, the CN@La@B + Light group showed the lowest bacterial survival rate (58.2%), whereas the remaining groups exhibited relatively weaker antibacterial activity. This result agrees with the in vitro antibacterial findings. In addition, these were no obvious differences in body weight among all groups during the 14-d treatment period (Fig. S19), suggesting that CN@La@B possesses acceptable biosafety with minimal systemic toxicity.

Histological evaluation of wound tissues collected on day 14 further supported the therapeutic effects of CN@La@B treatment. In H&E staining, compared with the PBS group, wounds treated with CN@La@B and CN@La@B + Light showed a more intact epidermis, along with continuous epithelial coverage and more regenerated skin appendages, including hair follicles and sebaceous glands (Fig. 6A and C). Analogously, Masson’s trichrome staining showed enhanced collagen deposition, with more densely packed and well-organized collagen fibers in the CN@La@B and CN@La@B + Light groups compared to the PBS group, suggesting improved extracellular matrix remodeling and structural integrity of the regenerated tissue (Fig. 6B and D). The inflammatory status of the wound tissues was further examined by immunofluorescence staining. As depicted in Fig. 6E to G, both CN@La@B and CN@La@B + Light groups displayed the reduced expression levels of IL-6 and TNF-α, proving accelerated resolution of inflammation. In contrast, strong cytokine-associated fluorescence remained visible in the PBS-treated wounds, suggesting that inflammation was still active at this stage. This reduced inflammatory response is likely owned to the release of BBR from CN@La@B, which regulates the wound immune microenvironment. Furthermore, angiogenesis was assessed by immunofluorescence staining of CD31 and VEGF. The CN@La@B + Light group exhibited enhanced neovascularization, with increased vessel density and elevated VEGF expression (Fig. 6H and Fig. S20), suggesting enhanced pro-angiogenic capability. Compared with other groups, more uniformly distributed and mature blood-vessel-like structures were observed, indicating efficient vascular remodeling. These findings suggest that CN@La@B, especially under visible-light irradiation, promotes infected wound healing by reducing bacterial burden, alleviating inflammation, and enhancing angiogenesis, thereby facilitating tissue regeneration.

**Fig. 6.**
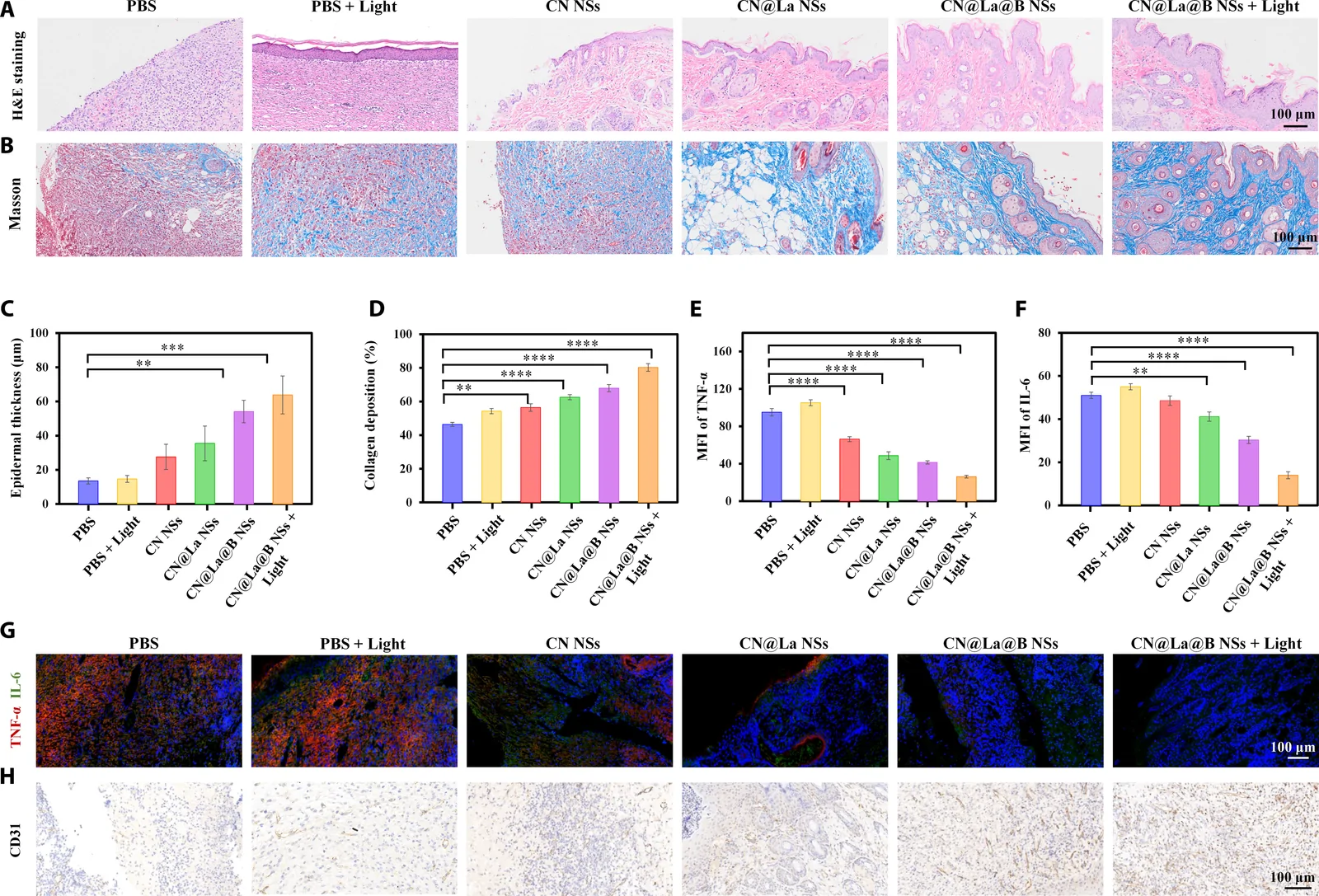
Histological and immunofluorescence analysis of infected wound tissues. (A) H&E staining and (B) Masson’s trichrome staining of wound tissues on day 14 following various treatments (scale bar: 100 μm). (C) Quantitative analysis of epidermal thickness by H&E staining (*n* = 3). (D) Quantitative analysis of collagen deposition by Masson staining (*n* = 3). Quantitative analysis of (E) TNF-α and (F) IL-6 expression by immunofluorescent staining (*n* = 3), with representative immunofluorescence images shown in (G) (scale bar: 100 μm). MFI, mean fluorescence intensity. (H) Immunohistochemical staining of CD31 under various treatments on day 7 (scale bar: 100 μm). For (C) to (F), **P < 0.01, ***P < 0.001, ****P < 0.0001.

## Conclusion

In this work, we applied a “flypaper-mimicked” antibacterial strategy to construct a multifunctional platform (CN@La@B) by immobilizing dendritic mesoporous La NPs onto g-C_3_N_4_ nanosheets and loading the anti-inflammatory agent BBR for infected wounds. The CN@La@B exhibited efficient bacterial capture capability, which could be due to the spiky topological La NPs that adhered to bacterial surfaces and induced phospholipid dephosphorylation, thereby enhancing membrane permeability. Meanwhile, visible-light-induced ROS generation was mediated by the CN@La heterostructure, where photoexcited electrons in g-C_3_N_4_ were transferred to La(OH)_3_, promoting electron–hole separation and ROS generation that further eradicated bacteria. BBR loaded on the platform simultaneously modulated the wound immune microenvironment by down-regulating pro-inflammatory cytokines. In vitro, CN@La@B killed bacteria (especially under visible light) and reduced LPS-induced inflammatory responses, while keeping both fibroblasts and RAW264.7 macrophages highly viable. In vivo, treatment with CN@La@B sped up wound closure, decreased bacterial load, reduced inflammation, promoted collagen formation, and stimulated new blood-vessel growth, especially under visible light. These results highlight CN@La@B as a multifunctional platform that synergistically integrates bacterial capture, membrane sensitization, ROS-mediated antibacterial activity, and immunomodulation, representing a non-antibiotic strategy for infected wounds.

Notably, visible-light irradiation suffers from limited tissue-penetration depth. Wounds covered by blood clots, thick exudates, or overlying soft tissue may substantially attenuate incident photons and compromise photocatalytic activation. Therefore, the present strategy is primarily applicable to superficial open wounds. Debridement to remove wound exudates and scabs may be required before light application in real-world clinical scenarios. Extending the excitation spectrum to the near-infrared region via rational band engineering may help overcome the limited tissue penetration of visible light and expand the potential application of this strategy to deep-seated bacterial infections. Further validation in clinically relevant models, including diabetic chronic wounds and polymicrobial biofilm infections, is warranted to recapitulate complex pathological scenarios. In addition, developing the nanoplatform into practical wound-dressing formulations and systematically evaluating its long-term biosafety and lanthanide biodistribution will be important for future translational development. This topology-mediated synergistic antibacterial paradigm may provide a promising strategy for the development of next-generation anti-infective nanotherapeutics.

## Ethical Approval

The animal experiments were carried out following the guidelines of the Shandong First Medical University and Shandong Academy of Medical Sciences for the Care and Use of Laboratory Animals and were approved by the Committee on Ethical Use of Animals of Shandong First Medical University and Shandong Academy of Medical Sciences (Ethical code: W202512290947).

## Data Availability

All data needed to support the conclusions in the paper are presented in the manuscript and/or the electronic supplementary material. Additional data related to this paper may be requested from the corresponding authors upon request.
