# Supplementary material for: Visible-Light-Enhanced Lanthanide Heterostructure with Synergistic Antibacterial and Anti-inflammatory Activity to Accelerate Wound Healing
Source: Biomater Res. 2026 Oct 21;30:0436. doi: 10.34133/bmr.0436 (PMC13683949; doi:10.34133/bmr.0436)
Supplement: Supplementary file 1 [file bmr.0436.f1.docx]

**Supporting Information**

**Visible-Light-Enhanced Lanthanide Heterostructure with Synergistic Antibacterial and Anti-Inflammatory Activity to Accelerate Wound Healing**

Ying Qu^1#^, Wenyu Zhang^1#^, Leilei Song^2^, Xinyu Wang^1^, Xinyi Zhang^1^, Kejian Wu^1^, Zhe Han^1^, Shuai Gao^4^, Huilin Zhao^3*^, Cong Song^1*^, Runxiao Zheng^1*^

^1^ Shandong Provincial Hospital, School of Public Health, Medical Science and Technology Innovation Center, Shandong First Medical University & Shandong Academy of Medical Sciences, Jinan, 250117, China.

^2^ School of Polymer Science and Engineering, Qingdao University of Science and Technology, Qingdao, 266042, China.

^3^ Shandong Medical and Pharmaceutical University, Yantai, 264003, China.

^4^ State Key Laboratory of Animal Biotech Breeding, College of Animal Science and Technology, China Agricultural University, Beijing, 100193, China.

.

^#^ These authors contributed equally to this work.

* Corresponding author. E-mail addresses: zhaohuilin@sdmpu.edu.cn (H. Zhao), [songcong@sdfmu.edu.cn](mailto:songcong@sdfmu.edu.cn) (C. Song) and [zhengrunxiao@sdfmu.edu.cn](mailto:zhengrunxiao@sdfmu.edu.cn) (R. Zheng).

**Figure S1.** Average particle sizes of CN NSs, La NPs, CN@La NSs or CN@La@B NSs determined from TEM images.

**Figure S2.** UV-vis calibration curve of BBR used for the determination of its loading capacity.

**Figure S3.** Determination of the release of La^3+^ from CN@La@B NPs by ICP-MS.

**Figure S4.** High-resolution XPS spectrum of N 1s for pristine CN@La@B.

**Figure S5.** High-resolution XPS spectra of O 1s for (A) pristine CN@La@B and (B) CN@La@B after phosphate adsorption.

**Figure S6.** High-resolution XPS spectrum of C 1s for (A) pristine CN@La@B NSs and (B) CN@La@B NSs after phosphate adsorption.

**Figure S7.** (A) TEM micrograph of as‑prepared La_2_O_3_ nanoparticles (La_2_O_3_ NPs), showing uniform smooth spherical morphology. Scale bar: 100 nm. (B) P Hydrodynamic size distributions NPs of the La and La_2_O_3_NPs. (C) Zeta potential of La and La2O3 NPs (n = 3).

**Figure S8.** SEM image of E. coli incubated with La_2_O_3_ nanoparticles. (scale bar: 500 nm).

**Figure S9.** Quantitative analysis of E. coli colonies. (n = 3).

**Figure S10.** Figure S10. Representative photographs of bacterial colonies grown on LB agar plates after various treatments.

**Figure S11.** Photographs of E. coli colonies on LB agar plates after different treatments.

**Figure S12.** Assessment of bacterial outer‑membrane permeability by N‑phenyl‑1‑naphthylamine (NPN) fluorescence assay (n=3).

**Figure S13.** Cell viability of L929 fibroblasts after treatment with different concentrations of CN NSs, La NPs, CN@La NSs, and CN@La@B NSs under visible light irradiation (n = 3).

**Figure S14.** Cell viability of RAW264.7 cells after treatment with different concentrations of CN NSs, La NPs, CN@La NSs, and CN@La@B NSs under visible light irradiation.

**Figure S15.** ELISA analysis of IL-6 levels in LPS-stimulated RAW264.7 cells under different treatments (n = 3).

**Figure S16.** ELISA analysis of TNF-α levels in LPS-stimulated RAW264.7 cells under different treatments (n = 3).

**Figure S17.** Photothermal heating curves recording the temperature variations of PBS, PBS + Light, CN NSs, CN@La NSs, CN@La@B or CN@La@B + Light under visible‑light irradiation (420–780 nm, 0.2 W/cm^2^).

**Figure S18.** Wound‑bed temperature‑variation curves for PBS, PBS + Light, CN NSs, CN@La NSs, CN@La@B, and CN@La@B + Light groups under visible‑light irradiation (420–780 nm, 0.2 W/cm^2^).

**Figure S19.** Body weight changes of mice in different treatment groups during the 14-day treatment period (n = 3).

**Figure S20.** Immunohistochemical staining images of VEGF on day 7 (scale bar: 100 μm).

**Materials and methods**

**Materials.** Dicyandiamide was purchased from Shanghai Macklin Biochemical Co., Ltd. (Shanghai, China). Citric acid and tetrahydrofuran were obtained from Sinopharm Chemical Reagent Co., Ltd. (Beijing, China). Berberine (BBR) was purchased from Macklin Biochemical Co., Ltd. (Shanghai, China). Sodium salicylate and lanthanum chloride hexahydrate were purchased from Shanghai Aladdin Biochemical Technology Co., Ltd. (Shanghai, China). The bacterial viability assay kit was obtained from Beyotime Biotechnology (Shanghai, China). Fetal bovine serum was purchased from Saiaomei Cell Technology Co., Ltd. (CellMax) (Beijing, China). MTT (Thiazolyl Blue Tetrazolium Bromide) was purchased from Biorioth Company (Beijing, China). Mouse TNF-α and IL-6 ELISA kits were purchased from BioLegend (San Diego, USA). All chemicals were of analytical grade and used without further purification.

**Synthesis of La_2_O_3_ nanoparticles (La_2_O_3_ NPs).** Take 10 mL of deionized water and add La_2_O_3_ nanoparticles to prepare a particle suspension with a final concentration of 50 μg/mL. Disperse the suspension using an ultrasonic device. Seal the well - dispersed particle suspension and incubate it in a constant - temperature environment at 37 °C for 24 h. After the incubation, transfer the suspension to a centrifuge tube and centrifuge it at 15000 rpm for 10 min. Discard the supernatant. Add fresh deionized water to the precipitate for resuspension, and centrifuge it again under the same conditions. Repeat the washing process 2 - 3 times to completely remove free impurity ions.

**General Characterization Techniques.** The morphology of the samples was characterized by transmission electron microscopy (TEM, HITACHI HT7800, Japan). The crystal structures were analyzed using an X-ray diffractometer (XRD, Bruker D8 ADVANCE/Ultima IV, Germany) at 8 °/min using Cu Kα radiation (λ=0.15405 nm, 40 kV, 40 mA). The elemental composition and chemical states were investigated by X-ray photoelectron spectroscopy (XPS, Axis Supra+, Kratos, UK). The zeta potential of the nanoparticles was measured using a zeta potential analyzer (Nano-ZS, Malvern, UK). Fourier transform infrared (FTIR) spectra were recorded on a Nicolet iS20 spectrometer (Thermo Fisher Scientific, USA) in the range of 4000-400 cm^-1^. UV-vis absorption spectra were obtained using a UV-vis spectrophotometer, and UV-vis diffuse reflectance spectra (DRS) were recorded to analyze the optical properties of the samples. All measurements were carried out at room temperature unless otherwise specified.

**Cell culture.** RAW264.7 macrophages were cultured in RPMI 1640 cell culture medium containing 10% fetal bovine serum (FBS), 1% Penicillin-Streptomycin at 37 °C in a humid atmosphere containing 5% CO_2_. The L929 murine fibroblast cell line (L929) was cultured in Dulbecco’s Modified Eagle’s Medium (DMEM) enriched with 10% FBS and 1% Penicillin-Streptomycin. All cells were maintained at 37 °C in a humidified atmosphere containing 5% CO_2_.

**Cell viability assay.** RAW264.7 macrophages (or L929 fibroblasts) were seeded in 96-well plates at a density of 5 × 10^3^ cells per well (100 μL per well) and incubated for 24 h to allow cell attachment. The cells were then treated with CN NSs, CN@La NSs, and CN@La@B NSs at different concentrations (0, 25, 50, 100, and 200 μg/mL) for 24 h. After treatment, MTT solution was added to each well and incubated for 3.5 h. The absorbance was measured using a microplate reader.
